# The feasibility and safety of ventral hernia repairs under local anaesthesia: a systematic review

**DOI:** 10.1186/s12893-025-02931-8

**Published:** 2025-05-26

**Authors:** Sarah Michael, Afifa Naseer, Munir Tarazi, Bhamini Vadhwana

**Affiliations:** 1 Department of General Surgery, Salford Royal Hospital, Northern Care Alliance, Salford, England, M6 8HD United Kingdom; 2 Department of General Surgery, West Hertfordshire Teaching Hospitals NHS Trust, Vicarage Road, Watford, England, WD18 0HB United Kingdom; 3 Department of Surgery and Cancer, Imperial College London, Hammersmith Campus, Du Cane Road, London, England, W12 0NN United Kingdom

**Keywords:** Hernia, Local anaesthetic, Ventral hernia

## Abstract

**Background:**

Ventral hernias represent a significant global healthcare burden. Repair under local anaesthesia (LA) provides benefits to patients, hospitals and economies. While inguinal hernia repair under LA has been established, this has not translated to other abdominal wall hernias. This systematic review evaluates the feasibility, safety, and efficacy of performing these repairs under LA.

**Methods:**

A systematic review was conducted using OVID^®^ EMBASE and MEDLINE to review articles published between 1966 and 2023. Thirty-three papers were included examining variables such as type of hernia, complications, cost-effectiveness, LA used and length of stay. All papers were quality assessed using the ROBINS-I tool. Papers assessing inguinal hernias were excluded.

**Results:**

13,491 patients underwent ventral hernia repair under LA. Complication rates for LA repairs are low, with wound infections and hematomas ranging from 0.3 to 2%. Recurrence rates were also low (0.3-2.5%). Early mobilisation and same-day discharge were notable benefits, with over 97% of patients ambulatory within hours. Postoperative pain was minimal, contributing to high patient satisfaction rates (90–97%). LA repairs proved especially beneficial for high-risk groups, including elderly and frail patients. However, these findings were only seen in hernia defects less than 5 cm. Heterogeneity among study populations, small sample sizes, and lack of standardisation in LA administration were noted.

**Conclusion:**

This review supports the broader implementation of LA for ventral hernia repairs in small defects (< 5 cm), demonstrating its safety, feasibility, and patient acceptability. Careful patient selection for standardisation of best practices for LA hernia repairs offers the potential for significant cost-savings with overall favourable outcomes.

**Supplementary Information:**

The online version contains supplementary material available at 10.1186/s12893-025-02931-8.

## Introduction

Hernias represent a significant healthcare burden worldwide and in the UK with over 100,000 hernia repairs performed each year [1]. Ventral hernias are a diverse group, constituting a large portion of these cases and are associated with various complications that can lead to significant morbidity, often necessitating surgical intervention.

Surgical intervention has become standardised with clear recommendations for the use of mesh and adequate overlap in both open and laparoscopic hernia repairs. However, there is no consensus on the best method of anaesthesia. Currently local anaesthetic (LA) repairs are limited to specialised hernia centres with general anaesthetic (GA) or regional anaesthetic remaining the favoured approach [2].

General anaesthetic repairs can limit the number of patients eligible for elective repair due to high-risk co-morbidities. Ventral hernia repairs under LA can provide a viable alternative. Studies have suggested that LA repairs may provide some benefits including reduced length of stay, financial benefits to the institution and increased patient satisfaction [3]. There are however concerns about the feasibility of its routine use due to increasing complexity of the surgery. Nevertheless, LA repair of inguinal herniae have been well established. Systematic reviews comparing the use of LA and GA approach in inguinal hernia repairs demonstrated greater patient satisfaction, reduced length of stay, reduced post-operative pain and nausea, and an earlier return to work [4, 5]. There has also been a previous systematic review focussing only on umbilical hernias but only had 9 articles included in the study and were unable to make any specific conclusions beyond feasibility [6]. The efficacy of the LA technique has been proven; however, its uptake has been limited with all types of ventral herniae, and no consensus has been reached on the feasibility of more widespread usage. There has been no previous systematic review to summarise the studies for local anaesthetic ventral hernia repairs.

The study aims are to evaluate the feasibility, complication rate, recurrence rate and patient satisfaction for ventral hernia repairs performed under LA.

## Methods

### Search strategy

A systematic search was employed using OVID^®^ EMBASE and MEDLINE databases to identify potentially articles for review published between 1966 to 5th March 2023. A search strategy formed with keywords and MeSH headings relating to “abdominal wall hernia,” associated hernia types and “local anaesthetic” used in combination with the Boolean operators AND and OR. Details of the full search strategy used is provided in Table 1. Eligibility criteria for inclusion was a study investigating any ventral hernia (epigastric, umbilical, paraumbilical, spigelian, incisional) except groin hernias repaired under local anaesthetic. This decision was made as inguinal hernia repair under local anaesthesia is an already established practice. Studies with only inguinal hernias in which no other data could be extracted were excluded. Studies with mixed types of hernia where data could be extracted were included. Case reports, editorial, opinions, reviews, commentary, conference abstracts, letters and non-English language articles were excluded.

### Data extraction

A total of 33 papers were included in this review for data extraction. 2 reviewers screened the initial papers and also to extract the data. Microsoft Excel was used for this process. The targeted variables for extraction were the type of hernia, number of patients having the procedure under local anaesthetic, age, gender (number of male patients), comparison group if applicable, type and volume of local anaesthetic used and the primary outcome i.e. clinical outcome, cost-effectiveness, quality of life, length of stay, complications. Two independent reviewers (AN, BV) screened all titles and abstracts to identify relevant articles for full text review.

### Outcomes

The primary outcome of this review was to reviewing complication rates, recurrence rates, patient satisfaction and to identify any clinical, patient or economic benefits in repairing ventral hernias under local anaesthesia, compared to the gold standard of general anaesthetic.

### Quality assessment

Quality assessment was performed using the ROBINS-I tool to assess the quality and risk of bias case-control and cohort studies across seven domains (AN); confounders, patient selection, classification of interventions, deviations from intended interventions, missing data, outcome measures and selection of reported overall result.

**Table 1:** Search strategy for systematic review

Searches in EMBASE	Results
1. “exp abdominal wall hernia”	43,752
2. “abdominal wall hernia.mp”	11,956
3. “exp umbilical hernia”	6547
4. “exp spigelian hernia”	596
5. “exp incisional hernia”	9132
6. “ventral hernia or umbilical hernia or paraumbilical hernia or para-umbilical hernia or para umbilical hernia or supraumbilical hernia or supra umbilical hernia or supra-umbilical hernia or incisional hernia or port site hernia or spigelian hernia.mp”	21,904
7. 1 or 2 or 3 or 4 or 5 or 6	52,915
8. “exp local anaesthesia”	51,278
9. “local anasesthesia.mp”	23,831
10. 8 or 9	65,110
11. 7 and 10	1187
Searches in MEDLINE	
1. “exp hernia, abdominal”	29,802
2. “abdominal wall hernia.mp”	847
3. “exp hernia, umbilical”	4034
4. “exp hernia, ventral”	10,993
5. “exp incisional hernia”	1335
6. “ventral hernia or umbilical hernia or paraumbilical hernia or para-umbilical hernia or para umbilical hernia or supraumbilical hernia or supra umbilical hernia or supra-umbilical hernia or spigelian hernia or port site hernia or incisional hernia.mp”	9786
7. 1 or 2 or 3 or 4 or 5 or 6	34,163
8. “exp anaesthesia, local”	18,247
9. “exp anaesthetics, local”	110,796
10. “local anasesthesia.mp”	13,320
11. 8 or 9 or 10	128,107
12. 7 and 11	993

## Results

**Fig. 1:**
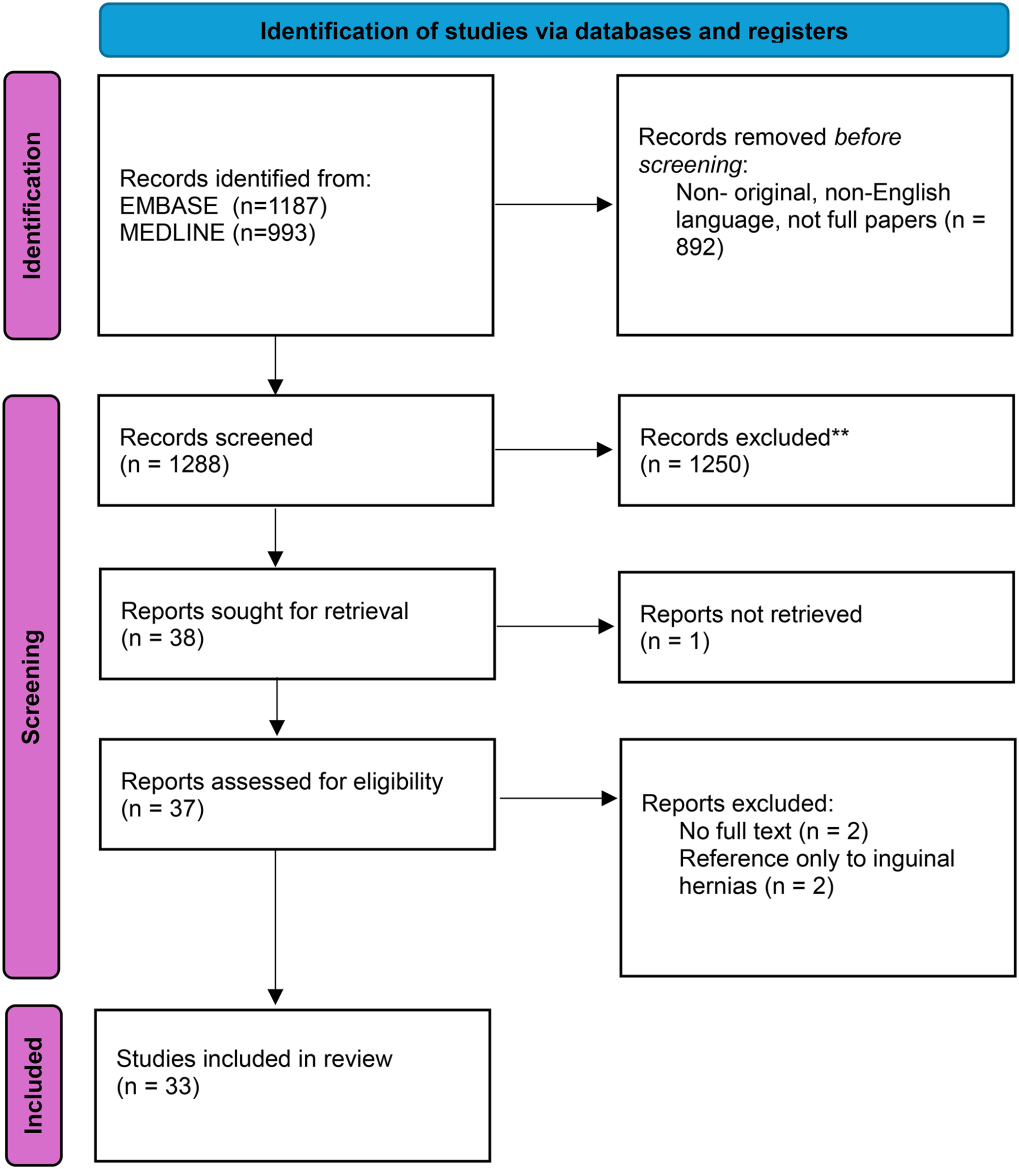
PRISMA flowchart detailing article selection

In total, 33 papers reported on 46,821 patients with 13,491 patients undergoing primary ventral hernia repair under LA, these were all in defects under 5 cm when mentioned. Figure 1 shows the PRISMA flowchart that details how the papers were selected. Hernias included in the review were those addressing all ventral hernias (*n* = 8), umbilical (*n* = 6), incisional hernias (*n* = 4), Spigelian (*n* = 4), a combination of inguinal, femoral and umbilical (*n* = 3), paraumbilical (*n* = 3), and the remainder reviewed a mixture of umbilical, epigastric and incisional (*n* = 5). Patient selection included cohorts of patients with frailty, high BMI, undergoing dialysis, patients who were pregnant, and those with minimal co-morbidities. Studies were produced from 12 countries, including the UK (*n* = 8), Italy (*n* = 8), Turkey (*n* = 3), the USA (*n* = 3), Spain (*n* = 2), Serbia (*n* = 2), Australia (*n* = 1), Chile (*n* = 1), Croatia (*n* = 1), India (*n* = 1), Argentina (*n* = 1) and Kenya (*n* = 1). 1 study was a prospective study while the remaining 32 papers were retrospective.

The type and volume of local anaesthetic used varied between each study. Twenty out of 33 studies (*n* = 61%) reported on composition and volume of LA used. The most common choice was a combination of lidocaine and bupivacaine [7–16]. The volumes administered were reported in 16 out of 20 studies (80%) with a range of 10-60mls [7–12, 15–20]. Three studies used sedation including midazolam, fentanyl and propofol [10, 12, 16]. There was no specific mention of intraoperative top-up of LA. The studies are summarised in Table 2.

Overall, 18 studies specifically assessed the safety and post-operative complication rates between 0.2 and 3%. Some studies reported complication rates as high as 20% however these were complications managed conservatively and in small study populations [21]. Across various hernia types, studies consistently reported low complication and recurrence rates. The follow up time for the studies ranged from 17 months to 168 months. Very few adverse effects were described with only one study reporting eight instances of intra-operative bradycardia which resolved spontaneously [22]. No anaesthetic complications were reported [22]. The majority of patients were discharged on the same day with only one outlier study reporting a length of stay of 3.5 days for undefined reasons [11]. Amongst special patient populations such as elderly (age 65–93) or obese patients there were similar complication rates and no perioperative mortalities reported [22].

Table 2 also includes studies that explored the feasibility of LA in novel techniques or special population groups including internationally [23, 24]. The use of LA in various techniques such as preperitoneal mesh and the darn technique [25–27] were found to be an effective anaesthetic method. Similarly, it was found to convey similar complication rates of 0.14% vs. 0.2% for haematomas for obese patients although longer operation times of 62 min vs. 78 were observed [14]. It has also been demonstrated to be cost effective in one study which reported a cost of almost half of a repair under GA in one hospital in Spain (€3270.37 for LA vs. €4740.37 for spinal vs. €7318.44 for GA) [28].

A high patient preference and high patient satisfaction rate for LA repair ranging from 90 to 97% in binary qualitative patient satisfaction surveys has also been reported [16, 20, 29–31]. These studies also highlight low self-reported perioperative and post-operative pain. When quantified in one study, 95% reported no pain contributing to overall positive patient experiences [31]. Only one study reported an increased preference for GA and this preference was reduced when considering patients who had previous experience of hernia repairs [29].

**Table 2:** Table summarising findings of studies assessing recurrence, complication rates and patient satisfaction of LA repair of ventral hernias

Author, year, country	Total number of patients	Number using LA *n* (%)	Hernia type	Local anaesthesia/sedation used	Recurrences	Complication	Patient satisfaction	Other findings
Herszage et al. 1999, Argentina	2100	2100 (100%)	All abdominal wall hernias	60-80mls 0.5% bicarbonated lidocaine with or without epinephrine	Not specified	0.3% complication rate. No hospital admissions required	Not specified	Early ambulation
Garcia-Urena et al. 2000, Spain	157	101 (64.3%)	Umbilical and epigastric	Max 60mls 1% lidocaine	2% recurrence rate	7.6% complication rate	Not specified	97% were discharged same day
Gianetta et al. 2004, Italy	16	16 (100%)	Umbilical and inguinal	Mepivacaine-Cl 2% and sodium bicarbonate 8.4% (volume not specified)	No recurrences	0% complication	Not specified	Patients undergoing peritoneal dialysis. Same day/early discharge and same day return to dialysis
Donati et al. 2008, Italy	29	29 (100%)	Incisional	Mepivacaine 2% and Bupivacaine 0.5% (volume not specified)	No recurrence	20% (seroma)	Not specified	All patients discharged within 7 h. Mild postoperative pain only.
Gianetta et al. 1997, Italy	232	232 (100%)	Umbilical and inguinal	Mepivacaine 2%, 0.5% bupivacaine, 8.4% sodium bicarbonate (volume not specified)	0.8%, 2 recurrences	2% haematoma, 1% wound infection rate.	Not specified	Elderly patients. No mortality.
Meier et al. 2021, USA	36,947	4958 (13.4%)	Umbilical	Not specified	Not specified	1.6%	Not specified	12–24% faster operative time
Zuvela et al. 2021, Serbia	476	476 (100%)	Umbilical, epigastric, incisional and spigelian	0.5% levobupivacaine, 2% procaine. 20mls and 30mls respectively and 50mls of saline.	2.5% recurrence rate overall.	1.9% of surgical site infection, 0.2% of superficial wound infection and 1.7% of deep mesh infection. 0.8% incidence of chronic pain.	Not specified	N/A
Donati et al. 2010, Italy	129	71 (55.0%)	Incisional	20mls solution A (7 ml 0.9% NaCl, 3 ml NaHCO2 and 10 ml Bupivacaine 2% without adrenaline), and 0-98mls of solution B (: 60 ml 0.9% NaCl, 7 ml NaHCO2 and 20 ml Bupivacaine 2%), 0-10mls Ropivacaine 10%.	2.8% recurrence rate.	Not specified	Not specified	97.2% of patients were immediately ambulated. Median operative time 101 min.
Ozyaylali et al. 2012, Croatia	10	10 (100%)	Inguinal, femoral, umbilical, and epigastric	IV midazolam and fentanyl. 1% lidocaine (14-30 ml) and 0.5% bupivacaine (10-30 ml)	Not specified	Low complication rate- number not specified	States “excellent patient acceptance”- no quantitative measure	N/A
Aureggi et al. 1998, Italy	468	468 (100%)	Inguinal and femoral	Not specified	Overall recurrence 1.1%	3.9%	Not specified	All patients immediately ambulated.
Dhumale et al. 2010, UK	1164	1164 (100%)	All abdominal wall hernias	Not specified	0.3% recurrence rate.	0.77%	Over 90% patient satisfaction rate.	No conversion to GA
Privitera et al. 2003, Italy	16	12 (75%)	Incisional hernia	Not specified	No recurrences.	Not specified	Not specified	All patients discharged within 24 h. Mild postoperative pain.
Malazgirt et al. 2003, Italy	4	4 (100%)	Spigelian	65 ml (range 40–90 ml) of saline solution including 1:3 bupivacaine (v: v) and 1/200,000 adrenaline (g: g)	Not specified	Not specified	Not specified	Average length of discharge 3.5 days. Return to normal activity after 9 days
Kulacoglu et al. 2012, Turkey	100	100 (100%)	Umbilical	2% lidocaine then 0.5% bupivacaine. Mean 33mls used. Midazolam, propofol and fentanyl used.	No recurrences.	One episode of chronic pain.	97% patient satisfaction.	Need for higher doses of LA with increased BMI, large defects, and recurrent hernias
Yang et al. 2021, China	23	23 (100%)	Ventral	40 ml mixture of 0.2% lidocaine, 0.01% ropivacaine, and 1/1,000,000 adrenaline and 10 ml normal saline was diluted with 160 ml normal saline.	No recurrence.	3 seromas and 1 surgical site infection	Not specified	Not specified
Zuvela et al. 2013, Serbia	8	8 (100%)	Spigelian	20 ml 0.5% levobupivacaine, 50 ml 2% procaine, and 30 ml saline solution	No recurrences	No complications	Not specified	Not specified
Kurzer et al. 2004, UK	54	49 (90.7%)	Umbilical	0.25% bupivacaine (volume not specified)	No recurrences	7 superficial wound infections	Not specified	Mild post operative pain
Menon et al. 2003, UK	32	32 (100%)	Umbilical	20mls 1% xylocaine in 1/200,000 adrenaline and 20mls 0.5% bupivacaine	No recurrences.	2 wound infections.	Not specified	Not specified
Acevedo et al. 2010, Chile	2031	2031 (100%)	All abdominal wall hernias	0.5% alkalinized Lidocaine with adrenaline. Volume not specified.	Not specified	0.3%	97.8% satisfaction	16 min average longer operation in obese patients
Ibañez et al. 2011, Spain	400	74 (18.5%)	Inguinofemoral, umbilical, epigastric and spigelian	Not specified.	Not specified	23 complications total- not specified for local or general	Not specified	Cost effectiveness- €3270.37 for LA vs. €4740.37 for spinal vs. €7318.44 for GA
Waweru et al. 2014, Kenya	239	48 (20.1%)	Inguinal, umbilical, epigastric and incisional.	Not specified.	Not specified	Not specified	Not specified	Feasibility of use internationally
Sadien et al. 2020, UK	123	123 (100%)	Paraumbilical	20 ml of 2% lidocaine with 1:200,000 adrenaline, 30 ml of 0.5% bupivacaine with 1:200,000 adrenaline, 50 ml of 0.9% sodium chloride and 6 ml of 8.4% sodium bicarbonate. Mean volume was 30.6 ml (range: 10.0–71.0 ml)	Not specified	Not specified	Not specified	Feasible in patients with raised BMI, no difference in doses of LA or in pain score
Buch et al. 2008, USA	12	12 (100%)	Umbilical and inguinal	Not specified.	No recurrence	No complications	Not specified	Feasibility in pregnant patients
Shah et al. 2021, India	95	Unknown	Umbilical, incisional, epigastric and paraumbilical	Not specified	Not specified	Not specified	Not specified	No specific finding related to LA. Highlights that it is feasible
Campanelli et al. 2004, Italy	32	32 (100%)	Spigelian	Not specified.	Not specified	Not specified	Not specified	Feasibility of preperitoneal mesh with LA.
Pawlak et al. 2021, UK	47	15 (31.9%)	Midline	Not specified.	Not specified	Not specified	Not specified	Feasibility of darn technique under LA
Senturk et al. 2016, Turkey	6	6 (100%)	Umbilical	Not specified.	Not specified	No minor or major complications	Not specified	Umbilical reconstruction can be performed under LA.
Licheri et al. 2004, Italy	7	3 (42.9%)	Spigelian	Not specified.	Not specified	Not specified	Not specified	Feasibility of prolene repair under LA
Gnanalingham et al. 1998, UK	75	75 (100%)	All hernias	Not specified.	Not specified	Not specified	33% preference for LA vs. 47% GA in total population. Those with previous hernia repair preferred local (28% vs. 23%)	Not specified
Loss et al. 2022, USA	449	53 (11.8%)	Umbilical	1% lidocaine with epinephrine (1:100 000) and one-fifth 4.2% sodium bicarbonate (2.5 mEq/5mL). 30mls volume used. Propofol, midazolam, fentanyl and ketamine used.	0 recurrence	6% complication rate. 13% for GA patients	Increased patient satisfaction. 94.7% would want further repairs under LA	Reduced operating time.
Bennett et al. 2013, UK	63	32 (50.8%)	Paraumbilical	20 ml 2% xylocaine with 1:200,000 adrenaline, 30 ml 0.5% bupivacaine with 1:200,000 adrenaline, 50 ml 0.9% sodium chloride and 6 ml 8.4% sodium bicarbonate.	Not specified	Not specified	Low perioperative pain, high satisfaction (96%).	N/A
Sinha et al. 2004, Australia	34	19 (55.9%)	Paraumbilical	1% Xylocaine mixed with 8.4% sodium bicarbonate (9:1). Volume not specified.	Not specified	Two post operative complications.	97% patient satisfaction.	N/A
Naseer et al. 2023, UK	28	28 (100%)	All abdominal wall hernias	Not specified.	Not specified	None reported	90% patient satisfaction.	Less theatre and recovery time

### Quality assessment

**Fig. 2:**
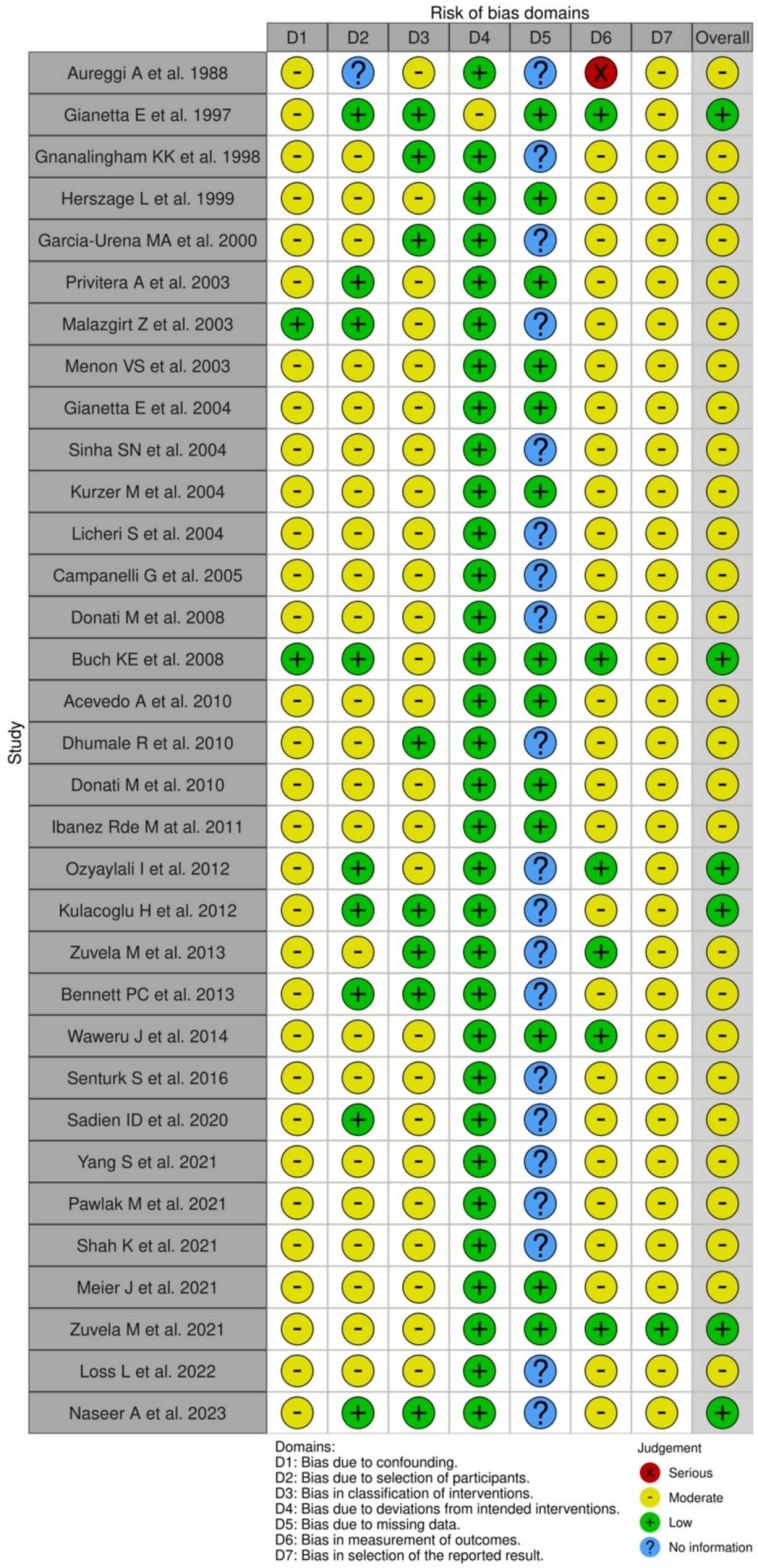
ROBINS-I tool for quality assessment of articles investigating the benefit of ventral hernia repair under local anaesthetic

The majority of the studies displayed a moderate risk for confounding factors (94%, *n* = 31), patient selection (70%, *n* = 23) and classification of intervention largely owing to its retrospective nature (76%, *n* = 25). There was a low risk of deviation from the intended intervention (97%, *n* = 32), with a moderate risk of outcome measures (79%, *n* = 26) and reporting results (97%, *n* = 32). Many studies did not report on missing data (61%, *n* = 20). Overall, 27 studies (82%) were considered at moderate risk of bias due to their retrospective nature. The summary of these results are shown in Fig. 2 which shows the quality assessment of each study using the ROBINS-I tool.

## Discussion

Overall, this study primarily demonstrates the feasibility, safety, and patient satisfaction for ventral hernia repair under local anaesthesia. The majority of studies reviewed showed local anaesthesia administered was effective, well tolerated by patients and associated with low complication and recurrence rates. While the efficacy of this has been previously well established for inguinal hernias, with one meta-analysis showing the superiority of LA versus all other types of anaesthesia, this has not yet been demonstrated for other abdominal wall herniae [3, 32, 33].

### Complications, recurrence and feasibility

Low complication rates were reported, with 0.3 to 2% rates of wound infection and haematoma formation [2, 7, 13, 17, 18], suggesting comparable outcomes to those performed under GA [34]. In one study, the complication rate for GA was almost double that of LA [16]. The most common complications recorded were superficial wound site infections, seromas, haematomas, 1.7% rate of mesh infections, with no observed mortalities. Chronic pain was reported in one case 3 months following surgery, however, the patient fully recovered after 1 year [12]. Recurrence rates were also low, ranging from 0.3 to 2.5%, with some studies reporting no recurrences [7, 17]. This is in keeping with the lower end of reported recurrence rates across all types of hernia repair, typically ranging from 0.5 to 15% [35–37]. One study did report a higher recurrence rate of 20% but this was only 3 conservatively managed seromas in a study population of 29 [21]. Dhumale et al. investigated 1164 patients who did not require conversion to GA, and achieved excellent outcomes [38]. However, these studies primarily included small patient numbers and targeted specific patient populations, often excluding recurrent hernias, large defects, or patients with significant co-morbidities. The size of the defects reported were up to 5 cm. Exclusion criteria across the studies encompassed individuals with high ASA grades, insulin-dependent diabetes, morbid obesity, as well as patients with large defects and associated obstructive symptoms. With stringent exclusion criteria, approximately two-thirds of all patients with ventral hernias may be eligible for surgical repair under LA [8]. Total time spent in theatre also varied with some studies reporting shorter operating and recovery times and others reporting comparable length of time to a general anaesthetic or longer in more complex patients [9, 14, 16, 31, 34].

### Pain and ambulation

The studies demonstrated early mobilisation, day case discharge and earlier return to normal activities. For example, 91% of patients (*n* = 891) in the UK undergoing an inguinal hernia repair, returned to normal activity and work after 5 days [39]. Early ambulation emerged as a common theme and an important outcome with over 97% ambulatory within hours and same day discharge [7–9, 21, 40–42]. A single study was an outlier by reporting an average length of stay of 3.5 days and a return to daily activity after 9 days in patients having preperitoneal mesh repair of spigelian hernias [11]. Although this is significantly longer than the aforementioned studies, it is still within the range described in the literature for open spigelian repairs which reports between 1 and 7 days [43, 44].

A trend in early mobilisation and discharge can be associated with low intraoperative and postoperative pain scores. Six studies reported low or negligible pain levels [20, 31]. Postoperative pain levels were also minimal with very few patients requiring regular analgesia on discharge. Patient-reported pain assessment methods, such as visual analogue scores, consistently indicated low levels of pain [10, 13, 21, 42].

### Feasibility amongst different populations and techniques

Studies have assessed the practicality of using LA for special population groups including dialysis patients, pregnant patients and the elderly and frail patients. Albeit a small sample size, Buch et al. demonstrated the safety of repairing umbilical hernias in pregnancy (*n* = 12) with no reported complications, recurrences or adverse outcomes to the births [45]. The safety profile was comparable between high and low BMI groups, with the only difference in longer operative times in the high BMI cohort [14]. There are conflicting findings of the volume of local anaesthetic required with some studies showing higher volumes in high BMI patients (*n* = 100), and others showing no difference in LA volume (*n* = 123) [12, 15]. This confirms there is not unanimity in the volume of dose of LA required for repair under LA. The wide variety of formulations and volumes of LA used means that there is no standard best practice that can be gleaned from this review. Studies using sedation are likely to be important confounders in interpreting intraoperative pain and not allowing for a true comparison to GA techniques.

Surgery under LA is particularly beneficial to elderly and frail patients with significant co-morbidities. A study of umbilical hernias repairs under LA in 7 elderly patients including emergency cases were associated with only 1% wound infection rates, 0.8% recurrences and no mortalities although this was a small sample size [22]. In addition, a large study of 4958 frail patients demonstrated 24% quicker surgeries and 86% fewer complications compared to GA [34]. Finally, one small study of 16 peritoneal dialysis patients undergoing LA repairs found no complications or recurrences with patients able to return to their scheduled dialysis without delay [40]. This demonstrates the legitimacy of using LA in special and complex patient groups with careful consideration.

Hernia repair with alternative techniques such as umbilical reconstruction, the use of preperitoneal mesh for spigelian hernias, and the darn technique for midline hernias, have all been demonstrated under LA, however with small sample sizes and limited follow-up data [46]. Further studies are needed to assess the feasibility of more complex techniques.

### Patient satisfaction and choice

Multiple qualitative studies have demonstrated an increased preference for LA procedures (94.7%) [16, 29], and patient satisfaction between 90 and 97% [12, 20, 30, 31]. There were no quantitative findings in any studies however and study numbers remain small. These findings show a majority are satisfied with the overall experience and outcome and would choose LA procedures again.

### Summary

This review reveals that amongst a range of studies, there are low complication rates, ranging from 0. 3 to 2% for wound infections and haematomas, these are comparable to those reported under GA. This is reported in defects up to 5 cm. Importantly, no mortalities were observed and only one instance of chronic pain reported which resolved within a year. The recurrence rates were also low ranging from 0.3 to 2.5%, these align with the lower end of recurrence rates seen across all hernia types [47]. These findings may be partially due to careful patient selection and expertise in the centres where these were performed. Some studies did report higher rates but mostly in small sample sizes and complications that were conservatively managed [8, 21].

One of the significant advantages of LA highlighted in the review is early mobilisation and same day discharge of patients with over 97% ambulatory within hours of their operation and most patients returning to normal activities within a few days. The low intraoperative and postoperative pain will have also contributed to this with minimal need for analgesia reported amongst numerous studies. However, there was no consensus reached on the type of anaesthesia used and some studies used additional sedative agents such as midazolam which may confound these results. Only one study reported a specific cost saving, and this aspect needs further investigation. This combined with early mobilisation and discharge of patients shows an advantage to LA repairs and is in keeping with previous studies [32].

The usage of LA in patients traditionally considered high risk also underpins its utility in a variety of patient groups. Patients with high BMI, dialysis patients and elderly patients all had favourable outcomes for example a 1% wound infection rate and no mortalities in elderly patients and no delay to return to treatment for dialysis patients [22, 40]. However, some of these studies have very small patient groups and did not have long follow up times.

High levels of patient satisfaction were also reported, with 90–97% of patients expressing contentment with their LA procedures albeit in studies with small sample sizes and no quantitative way of measuring this. Many patients were happy with LA, citing reasons such as fast recovery times and low postoperative pain, although this patient reported preference was not by patients who had undergone both, but it was based on their perceptions and was not quantitatively measured with the studies asking simple yes or no questions [16, 20, 29–31]. There was also one study in which patients expressed a preference for GA over LA which further impacts the conclusion that it is the preference for patients [29].

### Limitations and future

Firstly, significant heterogeneity exists among the study populations, spanning multiple countries, age groups, and co-morbidities. Most studies employed strict selection criteria, further limiting the study population. Studies that directly compared both LA and GA patients did not report the type of repair used or type of mesh which is a significant limitation on some outcomes including recurrence and post operative pain levels. This also makes the impact of the findings of this review more difficult to generalise to all hernia repairs. Secondly, the majority of studies had small sample sizes, which hinders widespread generalisations of the findings. Moreover, most study types were retrospective, focusing on elective patients leading to selection bias. The studies around patient satisfaction were all qualitative in nature and with no standardisation means the outcomes are potentially unreliable and the results cannot be grouped to reach a conclusion due to the significant heterogeneity.

Prospective studies with direct comparison to groups undergoing general anaesthetic procedures would enhance the studys robustness as well as randomised controlled trials. There was limited quantitative data available to conduct a meta-analysis. One significant limitation is the inability to isolate only ventral hernias from papers that included multiple types of hernia and further studies are needed to isolate outcomes from each different type of hernia and compare these directly to outcomes under GA. An important further area of study would be to compare different protocols for LA and be able to suggest a guideline to standardise this. There was a wide variability in the protocols each paper used.

## Conclusion

Overall, this systematic review has demonstrated that repair of some ventral hernias under LA is safe and feasible, applicable to multiple population groups and associated with low recurrence and complication rates in small defects less than 5 cm in size. Furthermore, patient satisfaction and pain levels have been reported to be in favour of LA repairs although only in qualitative studies. However, careful patient selection and consideration of technique and dosages are essential to ensure effectiveness. Repair under LA may not be suitable or applicable for all type of hernias and will be impacted by location, size and type of repair performed. Nevertheless, this study has collated multiple studies that support the more widespread consideration of LA hernia repairs for elective ventral repairs for defects up to 5 cm in size. Further prospective studies including randomised controlled trials are needed to make further conclusive statements on this topic.

## Electronic supplementary material

Below is the link to the electronic supplementary material.

Supplementary Material 1

## Data Availability

All data analysed during this study is included in the supplementary data file.
